# Aerosol droplet-size distribution and airborne nicotine portioning in particle and gas phases emitted by electronic cigarettes

**DOI:** 10.1038/s41598-020-78749-6

**Published:** 2020-12-10

**Authors:** Hélène Lalo, Lara Leclerc, Jérémy Sorin, Jérémie Pourchez

**Affiliations:** 1 Ingesciences, 1 Chemin des Arestieux, 33610 Cestas, France; 2 Mines Saint-Etienne, Univ Lyon, Univ Jean Monnet, INSERM, U1059 Sainbiose, Centre CIS, 42023 Saint-Etienne, France

**Keywords:** Biomedical engineering, Chemical engineering

## Abstract

The reliable characterization of particle size distribution and nicotine delivery emitted by electronic cigarettes (ECs) is a critical issue in their design. Indeed, a better understanding of how nicotine is delivered as an aerosol with an appropriate aerodynamic size is a necessary step toward obtaining a well-designed nicotine transfer from the respiratory tract to the bloodstream to better satisfy craving and improve smoking cessation rates. To study these two factors, recent models of EC devices and a dedicated vaping machine were used to generate aerosols under various experimental conditions, including varying the EC power level using two different types of atomizers. The aerodynamic particle sizing of the resulting aerosol was performed using a cascade impactor. The nicotine concentration in the refill liquid and the aerosol droplet was quantified by liquid chromatography coupled with a photodiode array. The vaporization process and the physical and chemical properties of the EC aerosol were very similar at 15 watts (W) and 25 W using the low-power atomizer but quite distinct at 50 W using the high-power atomizer, as follows: (1) the mass median aerodynamic diameters ranged from 1.06 to 1.19 µm (µm) for low power and from 2.33 to 2.46 µm for high power; (2) the nicotine concentrations of aerosol droplets were approximately 11 mg per milliliter (mg/mL) for low power and 17 mg/mL for high power; and (3) the aerosol droplet particle phase of the total nicotine mass emitted by EC was 60% for low power and 95% for high power. The results indicate that varying the correlated factors (1) the power level and (2) the design of atomizer (including the type of coil and the value of resistance used) affects the particle-size distribution and the airborne nicotine portioning between the particle phase and the gas phase in equilibrium with the airborne droplets.

## Introduction

Electronic cigarette (EC) users (known as “vapers”) are frequently former or current smokers^1^. A sufficient and constant delivery of nicotine is key to improving smoking cessation rates. Nicotine replacement therapy (NRT) is a medically approved treatment to take in nicotine by a method other than continued tobacco use for smoking cessation. The idea behind these products is to allow smokers to get nicotine into their bodies without having to inhale the toxic substances contained in tobacco smoke. Various NRT products are available including nicotine gum, tablets, lozenges, patches, nasal sprays, and buccal inhalers. Even if ECs are not considered to be medical devices, ECs seem to be a popular smoking cessation tool in countries such as France or England^1^.

ECs were introduced on the market a decade ago. Although they have evolved by increasing their complexity and capacity, the principle goal of all EC use remains the same. They are electrically powered devices enabling the release of airborne nicotine without any combustion process. Although ECs may have very different shapes and technologies, they are mainly composed of a mouthpiece (or drip tip), a tank containing a heating coil wrapped with a cotton wick and immersed in a liquid, and a battery supplying energy.

Unlike traditional cigarettes that involve tobacco combustion, ECs use heat to transform the liquid (known as “e-liquid”) into an inhalable aerosol. All ECs contain a small heating element that vaporizes a refill liquid to generate the aerosol. The wick plays a major role in the generation process because, when soaked by the e-liquid contained in the tank, it transports the e-liquid from the tank to the heater coil. Effective transport is mainly governed by capillarity, which depends on both the permeability of the wick and the viscosity of the e-liquid. Battery activation causes the coil to heat up (mainly by the joule effect) and vaporizes a small amount of e-liquid to generate the aerosol. The e-liquid used in EC devices is mainly composed of propylene glycol (PG) and (or) vegetable glycerin (VG); ratios vary from 100% PG to 100% VG. Other chemical compounds are generally added to the e-liquid formulation, such as nicotine and (in smaller quantities) flavor, water, and ethanol primarly^2–4^. The chemical composition of the aerosol produced by the EC depends on several parameters such as the delivered power, the ingredients of the e-liquid, or the puffing conditions, number of puffs, volume and duration, and features of airflow, among others^5–7^. The aerodynamic diameter of the airborne droplets generated by ECs has a noticeable impact on the nicotine delivery to the vapers. Indeed, the accurate determination of the airborne particle-size distribution is inherent to the prediction of aerosol deposition within the respiratory tract^8–11^. Thus, to obtain an accurate description of the quantity of nicotine or toxicants absorbed by the user, it is important to fully characterize the relation between EC emissions and (1) the aerodynamic diameter of the droplets it produces and (2) the chemical composition of the airborne particles in equilibrium with gas phases^12–15^. Although the aerosol properties and the distribution of nicotine between the gas and particle phases have been widely studied for conventional cigarettes, currently there are only a few investigations for ECs^11,16–20^. The physical and chemical compositions of the aerosol, as well as nicotine release, are highly dependent on the conditions of its generation^21,22^.

So far, ECs have been tested on smoking machines that were more or less adapted for the testing of vaping devices. In these specific cases, commercial EC batteries were used and variations can occur because a battery can discharge or provide a false reading of the applied power; therefore, if the power delivered varies from one puff to another, then the amount of e-liquid consumption also varies. To overcome these issues, we designed a vaping machine (which we named U-SAV, for Universal System for Analyzing of Vaping) to control all the physical parameters influencing the e-liquid vaporization: power, resistance value, flow rate, inhalation time, atomizer, and so on. With regard to the airflow profile, supplied power generation, and e-liquid consumption, we demonstrated that our vaping machine provided reproducibility and repeatability of results^23^.

This paper focuses on how the power supplied to both low-power and high-power atomizers in ECs influences the particle-size distribution in the aerosol and the portioning of emitted nicotine during the particle and gas phases. By using a vaping machine that enables the generation of fully controlled emissions in association with a cascade impactor, we were able to study the influence of the specific power applied to both low-power and high-power atomizers on the physical and chemical features of the emitted aerosol.

## Materials and methods

### Emissions generated from ECs

**Figure 1:**
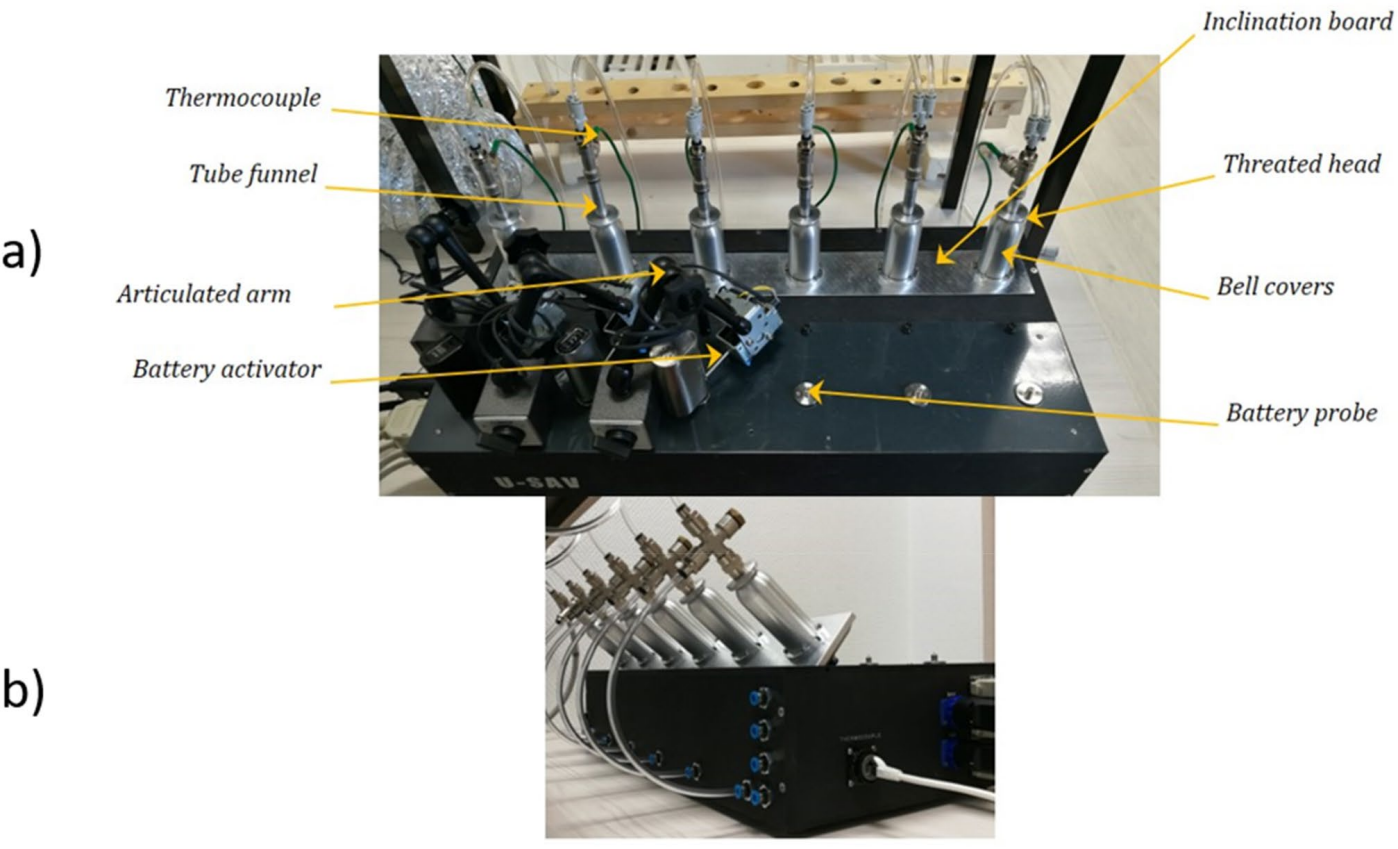
The universal system for analysis of vaping (U-SAV) machine. (**a**) The U-SAV machine allows the user to plug in a wide range of commercially available atomizers and batteries. (**b**) The atomizers can be tilted from 0° to 90°.

In this study, we used the U-SAV vaping machine (Fig. 1) because it allows the operator to fully control and monitor the physical vaporization parameters such as flow rate, power, resistance, inhalation time, and so on. All these parameters are monitored in real time so that the operator may record them and notice any abnormalities. The use of the U-SAV vaping machine allowed a constant power (15, 25, or 50 watts (W)) to be supplied to the atomizer during each experiment. Two different atomizers from a third generation of ECs were used according to the manufacturers’ instructions. The CUBIS atomizer from JOYTECH with a 1-Ω coil (using stainless steel wire of 6 cm length) was used at the 15 W and 25 W power levels. The atomizer TFV8 Baby from SMOK was used at the 50 W power setting with a 0.4-Ω V8-Baby Q2 dual core coil (double vertical coil using kanthal wire of 5 cm length for each coil). Coils were regularly changed during tests to ensure the proper continuity of the experiment. However, this experimental design introduced a confounding variable: the low-power (15 W and 25 W) and high-power (50 W) conditions required two different types of atomizer technologies and, therefore, the atomizer designs, resistance values, and other characteristics varied between them. Therefore, it was impossible to exclude a possible effect of the atomizer technology when the effects of the varying power levels were observed; however, we assumed that both power level and atomizer technology are always correlated.

The main focus of this work is to investigate the physical and chemical properties of the aerosol from ECs aerosol in real-life conditions. In this context, it may not be relevant for this paper to attempt to determine whether the differences in the aerosol are due to an increase in power or a change in atomizer technology when we interpreted results obtained from low-power or high-power conditions. This point is doubtless a main limitation, the differences in the aerosol are induced by the change of a pair of correlated factors (on the one hand the power level, and on the other hand the atomizer technology associated with a given resistance value) rather than the change of only one parameter. Indeed, we consider that the primary objective of this study is to highlight the physical and chemical differences in the generated aerosol for the two conditions: (1) aerosol generated at low power using low-power atomizers with a 1-Ω resistance and (2) aerosol generated at high power using high-power atomizers with an ultra-low resistance. To determine more precisely whether these physical and chemical characteristics of the aerosol observed during low-power or high-power aerosol generation can be generalized regardless of the type of atomizer, specific studies may be carried out in the future to compare different technologies that allow for high or low power to be generated.

Both of the atomizers have a refill liquid capacity of 2.5 mL but were only filled to 2 mL to avoid overfilling. The e-liquid used was 50% propylene glycol (PG) and 50% vegetable glycerin (VG) and a nicotine concentration of 18 mg/mL.

**Figure 2:**
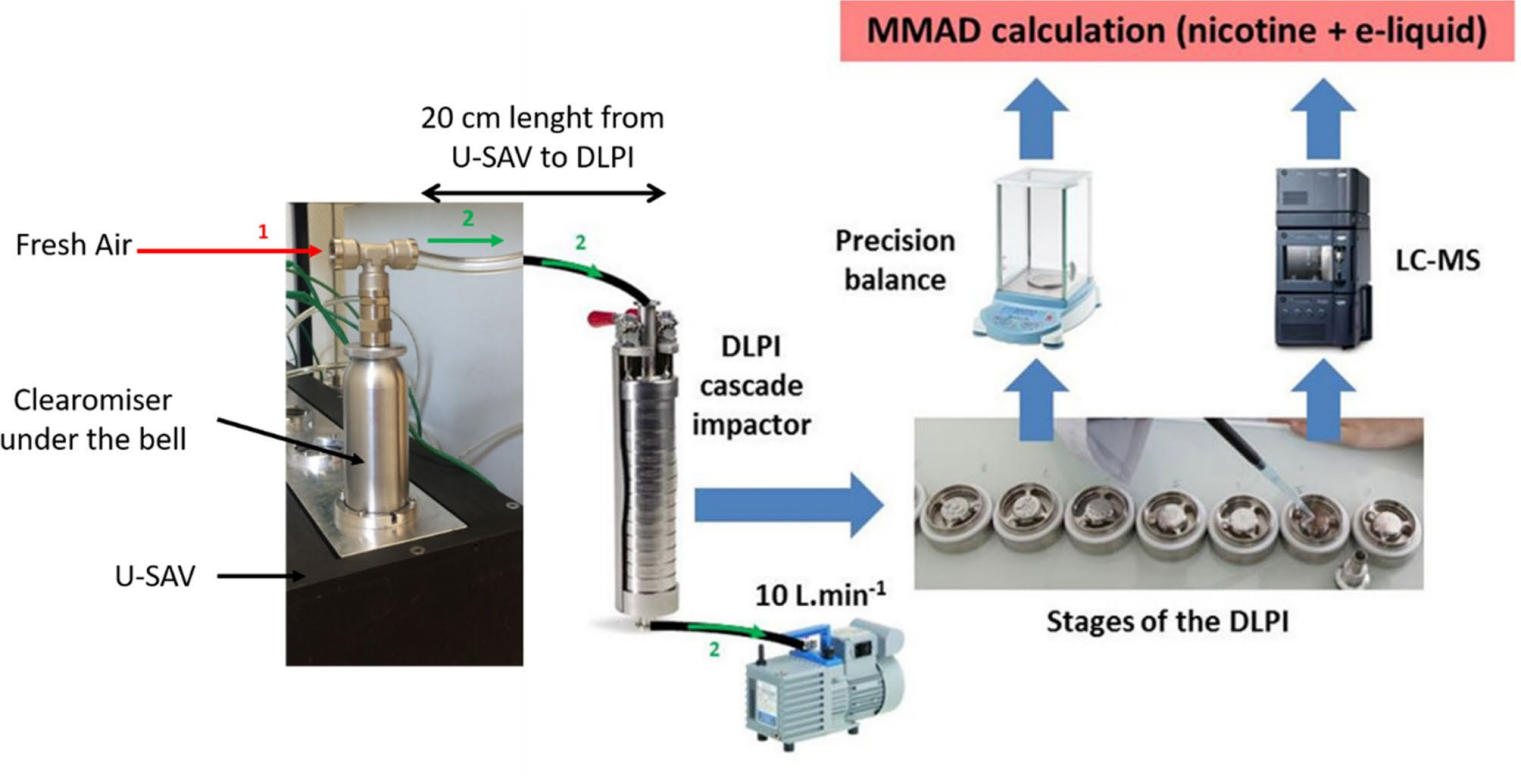
The experimental set-up for determining the particle-size distribution of aerosol generated by an electronic-cigarette. The emissions were generated by the U-SAV vaping machine and the particle-size distribution was measured by collecting and classifying the particles using a DLPI cascade impactor. The e-liquid collected on each stage of the cascade impactor was analyzed for nicotine concentration and density. Red arrow (1) indicates path of fresh air into the U-SAV machine. Green arrow (2) indicates path of aerosol particles through tubing from the U-SAV machine to the cascade impactor. *cm* centimeters, *DLPI* DEKATI low pressure impactor, *LC–MS* liquid chromatography–mass spectrometry, *L/min* liters per minute, *MMAD* mass median aerodynamic diameter, *U-SAV* universal system for analysis of vaping.

For all experiments, a delay was allowed between filling the atomizer and vaporization to completely soak the mesh and thus avoid dry burn (and thus false results). The atomizer was carefully shaken before each manipulation to mix the e-liquid properly without the generation of air bubbles. Then the atomizer was plugged into the U-SAV machine. Once the vaporization parameters were verified (resistance and power value, airflow), the U-SAV was connected to a cascade impactor (Fig. 2; see next section). To avoid vapor condensation, the flexible tube connecting the U-SAV machine to the cascade impactor was as short as possible (20 cm). Additionally, the airflow ring was always open to the maximum to let air pass through the atomizer. The inhalation time was set at 3 s and two different protocols were applied, according to the selected power level: (1) three puffs of 3 s with 27 s of rest between them for the 15-W and 25-W power levels, (2) two puffs of 3 s with 27 s of rest between them for the 50-W power levels (only two puffs were needed to avoid an overload of the cascade impactor stages).

### Particle-size distribution

Multistage cascade impactors are efficient tools to characterize relevant factors such as the particle-size distribution in aerosols. Cascade impaction is the method specified to obtain regulatory approval of inhalation products in the pharmaceutical sector^24^. This technique allows for the discrimination of the different components of the aerosol generated by an EC: matrix (PG, VG) or nicotine (active ingredient). Particle size is a key factor in the effective delivery of nicotine through the aerosol. Indeed, the effectiveness of nicotine depends on the aerodynamic diameter of the particles so that they are deposited in the ideal site in the lungs for absorption into the bloodstream. The best instrument to collect and sort the particles by size is a cascade impactor. On the basis of previous work^18,19^, we chose the DEKATI Low Pressure Impactor (DLPI), which (1) allows for the collection and classification of atomized particles ranging in size from 7 nm to 10 µm, which were divided into 12 fractions; and (2) operates with an airflow of 10 L/min, which is the required flow rate according to the operating instructions.

The aerosol particle sizes were measured in terms of their mass median aerodynamic diameter (MMAD). To obtain these measurements, the DPLI employed a 12-stage cascade low-pressure impactor, which determines (1) the gravimetric particle-size distribution, using an electronic precision balance (METTLER AT261 Delta, with a precision of 0.1 mg); and (2) the nicotine concentration (free-base or protonated forms), using Ultra-Performance Liquid Chromatography technology coupled with a Photo Diode Array detector (UPLC-PDA; WATERS).

To correctly use the DLPI and mimic the vaper’s behavior as closely as possible, the air-inlet mode was used on the U-SAV machine (that is, the air was forced before the atomizer) at 1.1 L/min, as described in Association Française de Normalisation (AFNOR) standard for emission generation^19^. The use of a vacuum pump ensured that the EC aerosol passed through the DPLI at 10 L/min (Fig. 2). As the EC aerosol was generated by the vaping machine with an airflow of 1.1 L/min, a connector placed on the top of the cover bell of the vaping machine enabled a fresh-air intake for dilution (Fig. 2). The tube connecting the U-SAV to the cascade impactor was 20 cm long, which allowed a delay (less than 1 s) between the generation of the aerosol and its passage to the first stage of the DPLI. Experiments were performed five times for each power level (15 W, 25 W, and 50 W). Results are expressed as an average of the five measurements ± standard deviation.

### Evaluation of nicotine concentration

Before each experiment, the 12 stages of the DLPI were weighed, allowing gravimetric monitoring. After each replicate, the DLPI was disassembled and all stages were weighed again for gravimetric size distribution. Then, to transfer the nicotine in the liquid phase collected by the DLPI, the DLPI stages were rinsed with 2 mL of methanol (HONEYWELL), and the flushing solution was kept in a volumetric flask. The chromatographic analysis for nicotine concentration was conducted with an Acquity UPLC-PDA using a CORTEC C18+ column (WATERS). The mobile phase was a mix of (1) distilled water containing 0.1% formic acid and (2) methanol of ultra gradient grade purity. The flow rate is set up at 0.7 mL/min.

Calibrations of nicotine range were made with a nicotine standard from Sigma Aldrich. A 3-point calibration was performed from 1 to 20 mg/mL nicotine (i.e. 20 ± 0.1 mg/mL, 10 ± 0.1 mg/mL and 1 ± 0.005 mg/mL), with five replicates performed at low calibration range to determine the LOD of 0.26 mg/mL and the LOQ of 0.39 mg/mL. All flushing solutions were kept at − 20 °C until analysis. The system control and data acquisition were performed using Empower 3 software (WATERS).

Finally, the nicotine concentrations of the aerosol droplets (the nicotine concentrations of the liquid phase collected by the DLPI cascade impactor) were calculated by dividing the total mass of nicotine (as determined by LCMS) by the total mass (or the volume, if the density was known) of the liquid aerosol droplets (as determined gravimetrically of the DLPI impaction plates).

## Results

### Effects of power on MMAD and e-liquid collection

**Figure 3:**
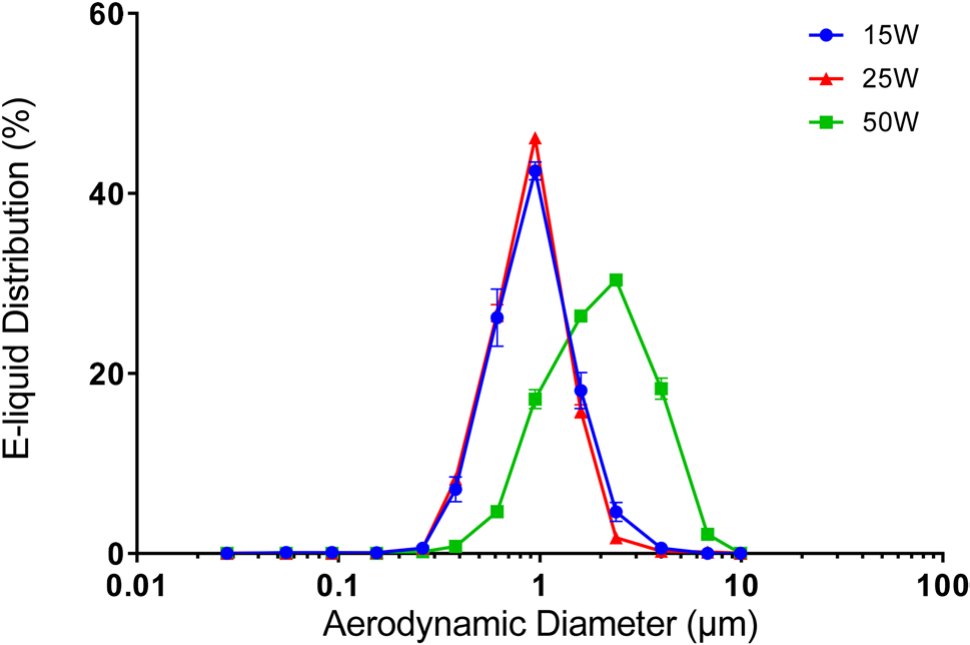
DLPI-collected data showing the effect of varying the power level on the frequency mass distribution of the e-liquid in an electronic cigarette. The results using the 15/25-W conditions (using low-power atomizer technology), and 50-W conditions (using high-power atomizer technology) are represented by a dot, triangle, and square, respectively. Experiments were carried out with an e-liquid composed of 50% propylene glycol and 50% vegetable glycerin with a nicotine concentration of 18 mg/mL (n = 5). *DLPI* DEKATI low pressure impactor, *µm* micrometers, *mg/mL* milligrams per milliliter, *W* watts.

Figure 3 indicates the mass distribution of vaporized e-liquid versus the aerodynamic diameter. In the 15/25-W conditions (using the low-power atomizer technology), a homogeneous repartition of e-liquid in the different particle sizes is observed (from 0.382 to 1.6 µm) with the majority in the 0.949 µm stage. For the 50-W condition (using the high-power atomizer technology), the aerosol particle size rises (from 0.613 to 6.8 µm) with the majority in the 2.39 µm stage. Table 1 indicates the aerodynamic particle sizing (that is, the MMAD and geometric standard deviation (GSD) of the size distribution) for the three tested power levels. Both Fig. 3 and Table 1 clearly demonstrate that the pair of correlated factors (power level and atomizer technology) affects the particle-size distribution since a difference between the 50-W condition and the 15/25-W conditions was observed. For 15/25-W conditions using the low-power atomizer technology levels, the results of the MMAD measurements were similar: 1.19 ± 0.01 µm for 15 W and 1.15 ± 0.01 µm for 25 W. However, when MMAD measurements for the 15/25-W and 50-W conditions were compared (corresponding to the change in atomizer technology from low-power to high-power), there was a noticeable increase in the MMAD: 1.15 ± 0.01 µm for 25 W and 2.46 ± 0.04 µm for 50 W.

**Table 1:** Summary of the particle-size distribution data.

Parameter (unit of measurement)	Power level, number of puffs, and atomizer technology		
	15 W (3 puffs) using low-power atomizer (n = 5)	25 W (3 puffs) using low-power atomizer (n = 5)	50 W (2 puffs) using high-power atomizer (n = 5)
Mass of e-liquid^a^	MMAD (µm): 1.19 ± 0.01 GSD: 1.62 ± 0.06	MMAD (µm): 1.15 ± 0.01 GSD: 1.56 ± 0.02	MMAD (µm): 2.46 ± 0.04 GSD: 1.93 ± 0.13
Mass of nicotine^b^	MMAD (µm): 1.06 ± 0.05 GSD: 1.53 ± 0.01	MMAD (µm): 1.12 ± 0.09 GSD: 1.48 ± 0.00	MMAD (µm): 2.33 ± 0.03 GSD: 1.71 ± 0.03

± corresponds to the standard deviation of the five experiments for each condition.

*GSD* geometric standard deviation of the mass aerosol distribution, *MMAD* mass median aerodynamic diameter, *µm* micrometers.

^a^Calculated using the mass of e-liquid collected on impactor stages.

^b^Calculated using the mass of nicotine collected on impactor stages.

The experiments also showed that an increase in power level led to an increase in e-liquid consumption by the EC: 8.3 mg/puff for 15 W, 11.7 mg/puff for 25 W, and 22.5 mg/puff for 50 W (Table 2). The e-liquid consumption is particularly marked at the transition between 25 and 50 W, corresponding to the change in atomizer technology from low-power to high-power. Indeed, the higher power implies a faster increase in the temperature of the coil, which involves (for the same amount of e-liquid composition inside the tank) increased consumption during the puffing experiments. Nevertheless, we emphasize again that the power level and atomizer technology are correlated factors. Indeed, because the wide range of power levels (from 15 to 50 W) can only be reached using two different atomizer technologies, we concluded that the difference in e-liquid consumption between the 25 and 50 W power levels was at least partially due to the atomizers used at each power level (Supplementary Information 1).

**Table 2:** Summary of consumption data.

Parameter (units of measurement)	Power level and atomizer technology		
	15 W using low-power atomizer	25 W using low-power atomizer	50 W using high-power atomizer
Mass of e-liquid consumed by EC during experiments (mg/puff)^a^	8.3	11.7	22.5
Mass of airborne e-liquid (mg/puff)^b^	7.9	11.6	13.3
E-liquid recovery rate (weight percent)^c^	95	99	59
Concentration of nicotine in the liquid particle phase (µg/puff)	88.5	130	230
Nicotine portioning in the particle phase (weight percent)^d^	62	64	NA^e^
Nicotine concentration in airborne droplets^f^ (mg/mL)	11.2	11.2	17.3

The recovery rate was calculated according to the e-liquid consumption that was used as a reference.

*DLPI* DEKATI low pressure impactor, *EC* electronic cigarette, *µg* micrograms, *mg/mL* milligrams per milliliter.

^a^Tank mass measurement.

^b^DLPI stage mass measurement.

^c^Ratio of e-liquid collected in the DLPI set-up compared to the e-liquid loss by EC.

^d^Sum of the nicotine dosage for each DLPI stage using the mass of airborne e-liquid calculated in footnote b.

^e^NA, Not applicable because the nicotine portioning in the particle phase for 50-W conditions cannot be rigorously estimated (using the calculation method described in footnote d) from the liquid collected in the DLPI because the recovery rate was too low to ensure that all the liquid collected by the DLPI was the entire liquid particle phase emitted by the EC.

^f^Nicotine in airborne droplets compared to the total nicotine mass emitted by the EC.

Moreover, the recovery rate of e-liquid mass collected in the DLPI was much smaller under the 50-W power level than for the low-power levels: 59% recovery rate for the 50-W power level and 95–99% recovery rate for the 15-W and 25-W power levels. The decrease in the recovery rate was due to the condensation of airborne droplets inside the drip tip, flexible tube, and upper part of the DPLI. The passage of air from the DPLI’s vacuum pump cooled down the tube and the impactor.

### Impact of power level on nicotine distribution and recovery

**Figure 4:**
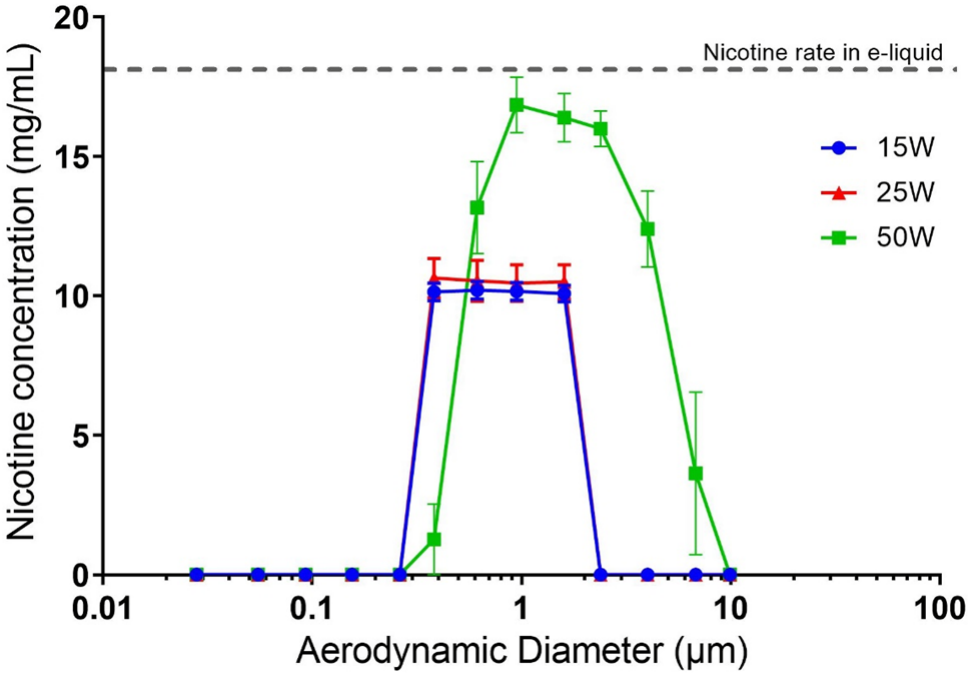
DLPI-collected data showing the effect of power level on the nicotine mass distribution. The results from using the 15 W, 25 W, and 50 W power levels are represented by a dot, triangle, and square, respectively. Experiments were carried out with an e-liquid composed of 50% propylene glycol and 50% vegetable glycerin with a nicotine concentration of 18 mg/mL (n = 5). *DLPI* DEKATI low pressure impactor, *µm* micrometers, *mg/mL* milligrams per milliliter, *W* watts.

Figure 4 displays the nicotine concentration in the e-liquid collected on impactor stages versus aerodynamic diameter. Compared to the initial nicotine concentration of 18 mg/mL initially introduced in the tank of the EC, we found a nicotine concentration in airborne droplets of approximately 11 mg/mL for 15 W and 25 W using the low-power atomizer technology, whereas this concentration reached approximately 17 mg/mL at 50 W using the high-power atomizer technology (Fig. 4). The nicotine concentration inside droplets of refill liquid, regardless of aerodynamic size, was constant for lower power levels (15 W and 25 W). However, there is clearly a distribution of concentrations shown for the 50-W conditions, demonstrating that the highest nicotine concentration within the aerosol droplets was found between 1 and 4 µm. This range suggests that most nicotine is found around the MMAD of 2.3 µm, but larger and smaller particles may have less nicotine delivery.

The results shown in Figs. 3 and 4 compared well with results from a previous study^18^. This tendency is confirmed by the similar MMAD values obtained in terms of airborne nicotine versus aerosol droplets: (1) 1.19 ± 0.01 µm versus 1.06 ± 0.05 µm for 15 W, (2) 1.59 ± 0.01 µm versus 1.12 ± 0.09 µm for 25 W, and (3) 2.46 ± 0.04 µm versus 2.33 ± 0.03 µm for 50 W (Table 1).

Finally, Table 2 shows a recovery rate of aerosol nicotine collected in the DLPI that was quite constant (around 60% of recovery) at lower power. The ratio of nicotine contained in airborne droplets collected in the DLPI stages compared to the total nicotine mass emitted by the EC clearly indicated that the nicotine portioning in the particle phase was quite constant in the 62% to 64% range for lower power. For the 50-W condition using the high-power atomizer technology, the low recovery rate of the e-liquid prevented the direct calculation of nicotine proportioning in the particle phase. However, by comparing e-liquid recovery and nicotine concentrations in airborne droplets, it was possible to hypothesize a quantity of nicotine in the particle phase for the 50-W conditions. Indeed, the nicotine concentration in the airborne droplets was close to the nicotine concentration in the e-liquid; therefore, we can assume that the nicotine portioning in the particle phase was close to 95%. Nevertheless, we again emphasize that the results do not mean that the DLPI collected 95% of the airborne nicotine for the 50-W conditions using the high-power atomizer technology. We showed that nearly 40% of the vaporized mass was lost under the 50-W conditions between the DLPI and the vaping machine (the bell of the U-SAV machine, drip tip, and 20-cm length of tubing). However, assuming that the nicotine was lost in a manner similar to the PG and VG and that the nicotine concentration in airborne droplets was close to the nicotine concentration in the e-liquid for droplets collected by the DLPI, it is reasonable to assume that the nicotine portioning in the particle phase was close to 95%.

## Discussion

The displayed results indicate the influence of the pair of correlated factors (1) the power level and (2) the atomizer technology on the particle size distribution and the airborne nicotine delivery. The only common point to all the power levels tested is that the MMAD of the aerosol droplets and MMAD of aerosol nicotine are quite similar. A good match is observed between profiles when the frequency mass distribution of the aerosol droplet is compared with the aerosol nicotine mass. This finding indicates the following: (1) there is no empty airborne carrier generated by the EC (that is, the droplet of refill liquid without nicotine); (2) the nicotine concentration inside droplets of refill liquid, regardless of the aerodynamic size, is constant for the lower power level; and (3) the particle-size distribution of the airborne refill liquid perfectly fits the particle-size distribution of the aerosol nicotine.

Besides, we demonstrated that for the 50-W condition using the high-power atomizer technology, the nicotine concentration in the airborne droplets was close to the initial nicotine concentration in the e-liquid. By contrast, for the 15/25-W conditions using the low-power atomizer technology, the nicotine concentration in the airborne droplets was significantly lower than the initial nicotine concentration in the e-liquid. The saturation ratio can be likely the cause for the higher aerosol nicotine concentration and the higher losses in the sample system at high power level. Indeed, for 50-W conditions, the high power device forces more mass of e-liquid into the vapor phase and created a warmer puff. The puff air saturates completely for PG, VG and nicotine. Thus, the aerosol forms condensation droplets in proportion to the source e-liquid, also the high vapor concentration is carried past the mouth piece into the tubing since it is warmer. Then, the cooling of the puff air causes condensation of the excess vapor. By contrast, at the lower power levels the puff air is not as saturated and does not deposit onto the tubing walls as much and does not fully condense. The saturation ratios for each component are dynamic but apparently quite similar at 15 and 25 W conditions compared to 50 W which is why the aerosol nicotine concentration is uniform and consistent at both lower power levels.

The experimental design introduced a confounding variable that is perfectly correlated with trials at 50 W, which is the use of a different atomizer. Indeed, the effects of varying the power levels can be evaluated by using two different atomizers, or by operating the atomizer below or above the manufacturer recommended range (but this last way will represent a limitation for applicability to realistic use representation). Thus, it is impossible to exclude the possible effects of the atomizer technology used during high-power conditions of 50 W compared to the low-power conditions of 15 W and 25 W.

Both the vaporization process and the physical and chemical properties of the produced aerosol are very similar at 15 W and 25 W using the low-power atomizer technology (MMAD of nicotine and e-liquid ranging from 1.06 to 1.19 µm, airborne nicotine concentration of approximately 11 mg/mL). However, for the 50-W condition using the high-power atomizer technology, an important change in the vaporization process is noticed with a rise of MMAD by a twofold factor and an increase of airborne nicotine concentration by a 1.5-fold factor (MMAD of nicotine and e-liquid ranging from 2.33 to 2.46 µm, airborne nicotine concentration of approximately 17 mg/mL). We can suppose that the role of coagulation on the growth of particle size is certainly critical. The growth rate can be extremely fast for particles at very high concentrations (i.e. before the aerosol passed through the tubing to the DLPI). This would contribute to explain a possible change of size of particles that exited the EC mouthpiece.

All things considered, the order of magnitude of MMAD obtained and the airborne nicotine concentration are in good agreement with previously published data^20,21^. In these publications, the authors find an MMAD at 13 W in ranging from 0.92 to 0.96 µm and of 1.10 µm at 22 W. These results correlate our findings. The slight difference for the experiment at 13 W can be explained by the protocol. Additionally, in the previous research, a syringe was used to transfer the vapor from the EC to the DLPI. In our research, the vapor was directly injected from the EC into the DLPI with a delay of less than 1 s but with a higher dilution ratio.

There seems to be a critical step in the EC vaporization process between the 25 and 50-W conditions that corresponds to the change from low-power to high-power atomizer technology (but also to the change from a resistance higher than 1 Ω to an ultra-low resistance), which significantly modifies the aerodynamic properties of the aerosol produced. It is important to note that the MMAD increases as well for the e-liquid mass as for the airborne nicotine concentration.

Our results indicate that the nicotine can be present in airborne e-liquid droplets but at a lower concentration compared to the initial nicotine concentration introduced into the tank of the EC (nicotine concentration of aerosol droplet at 11 mg/mL for 15 W and 25 W using the low-power atomizer technology and at 17 mg/mL for 50 W using the high-power atomizer technology, compared to 18 mg/mL in the e-liquid initially introduced into the EC). We conclude that there is a portioning of nicotine between the particle and the gas phases during the vaporization process for the 25–50 W transition (corresponding also to the transition between low-power and high-power atomizer technology). For the 15-W and 25-W conditions using the low-power atomizer technology, we found that approximately 60% of the total nicotine mass emitted by the EC was in the liquid droplet particle phase, whereas 40% of the total nicotine mass emitted by EC was in the gas phase in equilibrium with the airborne droplets. Although the gas phase was not directly studied in this specific case, it has to be taken into account for the total nicotine delivery. Indeed, during an experiment with a specific cryogenic trap, which allowed for the recovery of both particulate and gas phases, we generally recovered 100% of the expected vaporized nicotine.

Our results compare well to existing literature^19,25–29^. Several studies found EC aerosol particle sizes in the submicron size range (despite various protocols, sizing techniques, e-liquid compositions, and EC technologies); the majority of particles ranged from 0.172 to 0.5 μm in diameter in a study by Mulder et al.^25^; the dominant mode for the MMAD ranged from 613 to 949 nm in a study by Pourchez et al.^19^; and the median diameter of the EC particles ranged from 200 to 400 nm in a study by Zervas et al.^28^. Our results indicate a range of nicotine doses delivered in the liquid particle phase of 88.5–130 µg/puff for the low-power atomizer at 15 W and 25 W (Table 2), which compares well with a nicotine dose delivered in the aerosol between 88 and 125 µg/puff in a study by Peace et al.^26^.

**Figure 5:**
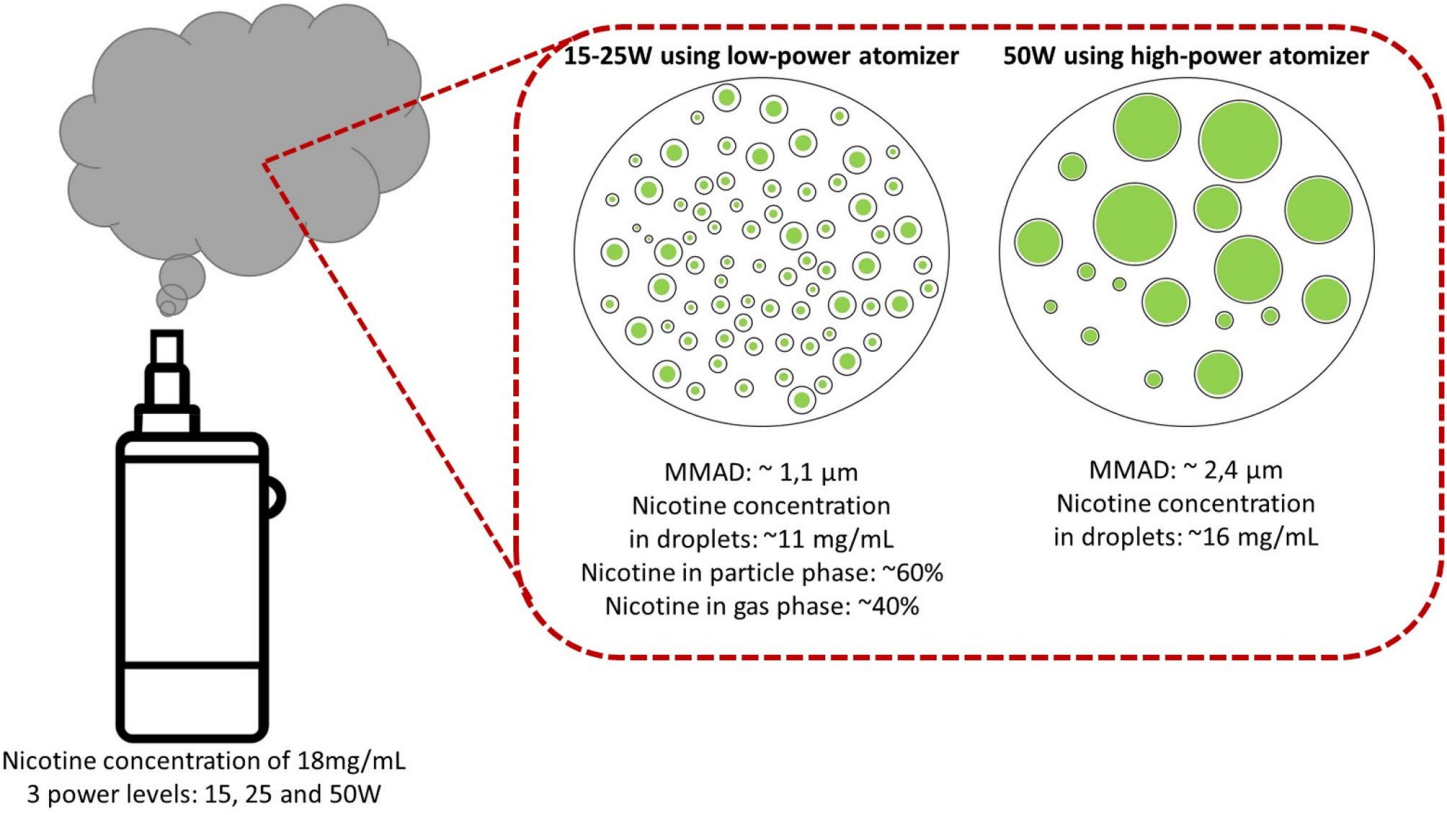
Portioning of nicotine in droplets of e-liquid during the vaporization process by power level (and, indirectly, by atomizer technology). The proportion of nicotine in the particle phase is obtained by a calculation that is based on the experimental data about e-liquid recovery and nicotine concentration in droplets. The result is an estimate by the research team and not a measurement. *MMAD* mass median aerodynamic diameter, *µm* micrometers, *mg/mL* milligrams per milliliter, *W* watts.

Figure 5 summarizes the results obtained using the low-power and high-power conditions. At 15 W and 25 W using the low-power atomizer technology, a homogeneous portioning of nicotine (nicotine concentration of aerosol droplets at 11 mg/mL) in the different particle sizes is observed (from 0.382 to 1.6 µm) with an MMAD of 1.1 µm. A portioning of 60% of the total nicotine mass emitted by the EC is present in the particle phase. By contrast, at 50 W using the high-power atomizer technology, the particle size increases (from 0.613 to 6.8 µm) with an MMAD of 2.4 µm. The concentration of nicotine in aerosol droplets is also higher (close to the tank concentration) under the high-power conditions compared to the lower power levels. Although it is not possible to rigorously measure the nicotine portioning in the particle phase (due to a low e-liquid recovery rate), we can assume by calculation and interpretation of results that almost all the nicotine is inside the droplet phase at 50 W. Therefore, we conclude that particle size and nicotine portioning in aerosol droplets are tightly linked to the power level of the EC. We interpret these results to indicate that a change in saturated vapor pressure, induced by a dilution of the EC aerosol, modifies both the particle size and nicotine concentration inside the droplets; that is, the partial evaporation of the EC aerosol, which is caused by a change in the saturated vapor pressure between low (15 W and 25 W) and high (50 W) power levels induces a decrease in the particle size and a decrease of nicotine concentration inside the droplets at the low-power levels.

## Conclusions

This paper highlights the strong influence of the power level and associated atomizer technology of the EC on the vaporization process of the e-liquid contained in the EC tank. An increase in power from low-power atomizer technology of 15 W and 25 W to the high-power atomizer technology of 50 W resulted in an increase in the quantity of e-liquid that was aerosolized. We conclude that there are no empty airborne carriers generated by the EC (that is, there are no droplets of refill liquid that do not contain nicotine) and that the nicotine concentration inside the aerosol droplets is constant for the low-power level (15 W and 25 W). We also noticed that the nicotine concentration of the airborne droplets is always lower than the nicotine concentration of the e-liquid that is initially introduced into the tank of the EC (approximately 11 mg/mL for 15–25 W and approximately 17 mg/mL for 50 W, compared to 18 mg/mL for the refill liquid).

At the low-power levels (from 15 to 25 W, using the low-power atomizer technology and a 1-Ω-resistance), the generated aerosol showed similar physical and chemical features (MMAD of nicotine and e-liquid ranging from 1.06 to 1.19 µm, airborne nicotine concentration of approximately 11 mg/mL). Our measurements indicated that approximately 60% of the total nicotine mass emitted by the EC was in the liquid droplet particle phase, whereas approximately 40% of the total nicotine mass emitted by the EC was in the gas phase in equilibrium with the airborne droplets.

However, for the 50-W conditions (using the high-power atomizer technology and ultra-low resistance), there is a step where the vaporization process changes with higher MMAD and airborne nicotine concentration (MMAD of nicotine and e-liquid ranges from 2.33 to 2.46 µm, airborne nicotine concentration of approximately 17 mg/mL). We assumed that approximately 95% of the total nicotine mass emitted by EC was in the liquid droplet particle phase.

On the basis of these results, we conclude that the higher the power level (using a specific high-power atomizer technology), the higher the (1) aerosol output, (2) MMAD, and (3) quantity of nicotine inside the aerosol droplets compared to the nicotine content in the gas phase.

## Supplementary Information

Supplementary Information.
