# Supplementary material for: Aerosol droplet-size distribution and airborne nicotine portioning in particle and gas phases emitted by electronic cigarettes
Source: Sci Rep. 2020 Dec 10;10:21707. doi: 10.1038/s41598-020-78749-6 (PMC7728817; doi:10.1038/s41598-020-78749-6)
Supplement: Supplementary file 1 — Supplementary Information. [file 41598_2020_78749_MOESM1_ESM.docx]

**Supplementary File**

**Aerosol droplet-size distribution and airborne nicotine portioning in particle and gas phases emitted by electronic cigarettes**

LALO Hélène^1^, LECLERC Lara^2^, SORIN Jérémy^1^, POURCHEZ Jérémie^2^

^1^ Ingesciences, 1 Chemin des Arestieux, 33610 Cestas, France.

^2^ Mines Saint-Etienne, Univ Lyon, Univ Jean Monnet, INSERM, U 1059 Sainbiose, Centre CIS, F - 42023 Saint-Etienne France.

**Evaluation of possible experimental biases induced by the use of low-pressure cascade impactor**

The first bias investigated was the possible evaporation phenomenon during the aerosol particle sizing due to the dilution occurring at low pressure inside the DEKATI Low Pressure Impactor (DLPI). To ensure that no experimental biases were induced by the evaporation of the propylene glycol and vegetable glycerin solvents or nicotine on the DLPI stages, control evaluations were conducted. Three types of tests were carried out for a total of 10 samples: a blank sample (an e-liquid simply diluted), a controlled sample where the DLPI stage was rinsed without any airflow, and a sample under experimental conditions (with an airflow of 10 liters per minute (L/min)). In the first case, the pure e-liquid was diluted to 1/20 with water (to simulate the rinsing of the DLPI stages). Then, the same quantity of e-liquid was poured (50 microliters (µL)) on each of the eight stages. Two stages were rinsed with only 950 µL of water, without airflow, and then were analyzed. Finally, the six other stages received an airflow of 10 L/min over 3 minutes, were also rinsed with 950 µL of water, and then analyzed.

Finally, to ensure that the correct vaporization of e-liquid occurred during the experiment and that neither the dilution nor the DLPI affected the quantity of e-liquid vaporized, control tests were conducted. Each of the three different material configurations was tested with the same protocol (airflow: 1.1 L/min, 3 second (s) per puff with 27 s of rest between puffs, three puffs for 15 watts (W) and 25 W, two puffs for 50 W), but without the dilution and the DLPI. The masses of the atomizers were measured before and after the vaporization. Each experiment was conducted three times.

**Evaluation of liquid evaporation**

On the basis of our investigations about the potential evaporation of liquid or nicotine on the DLPI stages, we demonstrated that there was no effect of the airflow on nicotine concentration (Supplementary Table 1). The concentration of nicotine remained the same, with or without airflow at 10 L/min.

**Supplementary Table 1.** Summary of evaporation data.

| **Sample** | **Nicotine concentration** |
| --- | --- |
| E-liquid | 18 mg/mL |
| Dilution to 1/20 | 1.1 mg/mL |
| Samples 1 and 2: no airflow | 1.1±0.02 mg/mL |
| Samples 3–8 + airflow at 10 L/min over 3 minutes | 1.07±0.04 mg/mL |

Abbreviations: L/min, liters per minute; mg/mL, milligrams per milliliter.

Note: n=2 for samples without an airflow, and n=6 for samples with an airflow.

**Evaluation of e-liquid vaporization**

In each of the nine cases (three different experiments conducted three times each), the same quantity of e-liquid was vaporized with or without DLPI and air dilution. The air dilution and the DLPI did not affect the quantity of e-liquid that was vaporized. Thus, we concluded that the vaporization process commonly occurs.

We investigated the potential impact of the airflow on nicotine or e-liquid evaporation. As shown in the supplementary data file, the results indicated that airflow did not affect the nicotine concentration. Indeed, the nicotine concentrations were almost the same for all three types of samples: control (simply diluted), rinsed on the DLPI stages without airflow, and rinsed on the DLPI stages with an airflow of 10 L/min.
